# The effect of non-steroidal anti-inflammatory drugs on the osteogenic activity in osseointegration: a systematic review

**DOI:** 10.1186/s40729-018-0141-7

**Published:** 2018-10-09

**Authors:** Jie Denny Luo, Catherine Miller, Tamara Jirjis, Masoud Nasir, Dileep Sharma

**Affiliations:** 1 College of Medicine & Dentistry, James Cook University, 14-88 McGregor Road, Smithfield, QLD 4878 Australia; 2 College of Public Health, Medical and Veterinary Sciences, James Cook University, 14-88 McGregor Road, Smithfield, QLD 4878 Australia

**Keywords:** Non-steroidal anti-inflammatory drug, Osseointegration, Osteoblast, Dental implant

## Abstract

Non-steroidal anti-inflammatory drugs are commonly used in implant dentistry for management of post-operative pain. The objective of this systematic review was to analyse the effect of non-steroidal anti-inflammatory drugs on the osteogenic activity of osteoblasts with an emphasis on its effect on osseointegration. A systematic literature search for in vitro, animal models, and clinical trials was conducted using Ovid, PubMed, Scopus, and Web of Science databases. Articles published since the introduction of selective COX-2 inhibitors, between January 1999 and July 2018, were selected. The integrated search followed the PRISMA statement with the following key terms: non-steroidal anti-inflammatory drug/s, titanium, osseointegration, and osteoblast. The review is registered at PROSPERO database: CRD42016051448. The titles and abstracts of each research article in the initial search (*n* = 875) were independently screened by two reviewers. A third independent reviewer reviewed the articles that were included by one but excluded by the other reviewer. This resulted in the cataloguing of 79 full-text manuscripts where the articles were assessed for the following criteria: the study investigates the effects of NSAIDs on osteoblasts, explores the COX pathway and its effect on osteogenic activity, and compares the effects of NSAIDs on osteoblasts with a control group. A total of 13 articles have been included for qualitative synthesis. There is a lack of consensus in the literature to explicitly conclude that there is a relationship between the use of post-operative NSAIDs and failed osseointegration; however, osseointegration does not appear to be negatively affected by NSAIDs in the human clinical studies.

## Review

### Introduction

Non-steroidal anti-inflammatory drugs (NSAIDs) are a group of drugs with anti-inflammatory, analgesic, and antipyretic effects. They are commonly used in dentistry for management of dental pain associated with inflammation. NSAIDs exert their effects through the inhibition of the cyclooxygenase (COX) enzyme, therefore interfering with the synthesis of prostaglandins (PG) and thromboxanes; PGs and thromboxanes are inflammatory mediators that are responsible for pain. COX has three isoforms: COX-1, COX-2, and COX-3. COX-1 exhibits characteristics of a constitutive enzyme, as its activity is associated with the involvement of PGs and thromboxanes in controlling normal physiological functions [1]. COX-2 exhibits characteristics of an inducible enzyme in inflammatory cells and is activated in response to pathological stimuli [2]. COX-3 is a variant of COX-1, though it shares the characteristics of both COX-1 and COX-2. The osseointegration process that is observed after implant insertion can be compared to bone fracture healing through the process of an inflammatory response in which the recruitment of osteoprogenitor cells occurs, followed by their downstream differentiation into osteoblasts that leads to bone deposition on the implant surface [3, 4]. COX-1 is expressed in normal bone and at the site of bone fracture, whilst COX-2 is upregulated during inflammation and the initial stages of bone repair [5]. The effects of NSAIDs on altering bone growth, remodelling, and repair are generally not considered when prescribed for post-operative pain management after implant placement.

Hypoxia occurs locally in bone tissues when pathological conditions such as implant placement and bone fractures arise [6, 7]. It has been established, through clinical studies of bone cultures, that hypoxia is directly responsible for directing the synthesis of prostaglandin E (PGE) by osteoblasts [6]. Therefore, the presence of the COX enzymes in bone healing is of importance [6]. Prostaglandins have the capacity to influence bone metabolism and can both induce and inhibit tissue repair mechanisms [6]. Local administration of PGE1 has been shown to stimulate bone formation, increase alveolar bone height, and induce formation of new cementum and periodontal ligament adjacent to the site of delivery in canine mandibles [8, 9]. Furthermore, PGE2 has been shown to stimulate replication and differentiation of osteoblasts cultured on smooth titanium surfaces thereby increasing bone formation around titanium implants [10]. Additionally, PGs can also inhibit the formation and growth of osteoblasts [6]. Therefore, altered PG levels as a result of COX inhibition can have a negative impact on the role of PG in bone tissue, potentially causing a shift in precursor cell action towards bone resorption [6].

Cyclooxygenases have an important role in the production of PGs where these enzymes in bone tissues show increased activity under the influence of hypoxia-inducible factors [6, 11]. Therefore, local activity of COX enzymes promotes bone formation and resorption through the production of PGs [12]. Non-selective NSAIDs are reported to inhibit the activity of COX-1 equally, if not more than COX-2 [2]. Therefore, NSAIDs inhibit the production of PGs at the site of implant placement or fracture, thereby influencing the bone healing cascade [13]. There is evidence from animal studies that indicate that COX-2 inhibitors delay done healing in diaphyseal fracture models in rats [13]. However, the exact roles of COX-1 and COX-2 in the PG production has not been ascertained in humans, and assumptions have been made suggesting a milder or non-significant inhibitory effect of selective COX-2 inhibitors on bone healing when compared to a non-selective COX inhibitor [2, 13]. Furthermore, a systematic review conducted by Marquez-Lara et al. highlighted the great variability regarding the impact of NSAIDs on bone healing, and that there is no consensus regarding the impact of NSAIDs following orthopaedic procedures [14]. Therefore, the rationale of the present systematic review is to address the gaps in the literature by identifying if variables such as the dosage, duration of administration, and selectivity of post-operative NSAIDs negatively affect osseointegration.

### Material and methods

#### Protocol and registration

The systematic review was conducted in accordance with the Preferred Reporting Items for Systematic Reviews and Meta-Analyses (PRISMA) statement [15]. The review is registered at PROSPERO database, and the review protocol can be accessed at http://www.crd.york.ac.uk/PROSPERO/display_record.php?ID=CRD42016051448.

The PROSPERO registration number is CRD42016051448.

#### Eligibility criteria

The review included in vitro, clinical and in vivo studies; animal models. Articles published since the introduction of selective COX-2 inhibitors in 1999 were included [3]. Studies published outside this time period, not in the English language, non-peer reviewed, and review studies were excluded.

#### Information sources

An electronic search into four databases: Ovid, Pubmed, Scopus, and Web of Science was performed to systematically identify the available literature. Articles published between January 1, 1999, and July 7, 2018, were considered.

#### Focus question

The focus question, used to guide the search strategy, according to the PICO schema is “Will variables such as the dosage, duration of administration, and selectivity of post-operative NSAIDs impair the bone healing around titanium implants?”

#### Search strategy

The search string comprised the combination of medical subject headings (MeSH) and free keywords. The linkage was conducted using the Boolean operator (AND, OR). The choice of keywords was intended to be broad to maximise the number of relevant studies considered. The following search strategy was applied to Ovid and PubMed:

1. (anti inflammatory agents, non steroidal[MeSH Terms]) AND osseointegration[MeSH Terms]
2. (anti inflammatory agents, non steroidal[MeSH Terms]) AND osteoblast[MeSH Terms]
3. (anti inflammatory agents, non steroidal[MeSH Terms]) AND dental implants[MeSH Terms]

Furthermore, the following search strategy was applied to Scopus and Web of Sciences to supplement records identified through Ovid and PubMed:

(non steroidal anti inflammatory agent OR non steroidal anti inflammatory agents OR non steroidal anti inflammatory drug OR non steroidal anti inflammatory drugs OR cyclooxygenase inhibition OR COX inhibition OR ibuprofen OR celecoxib) AND (osseointegration OR osteoblast OR osteoblasts OR titanium implant OR titanium implants OR dental implant OR dental implants)

#### Study selection

The titles and abstracts of each research article in the initial search were independently screened by the primary (JDL) and the second reviewer (TJ). A third independent reviewer (MN) reviewed the articles that were included by one but excluded by the other reviewer. The full-text manuscripts of the articles were catalogued in accordance to the “Eligibility criteria” section as mentioned above and were assessed for the following criteria:

- The study explored the COX pathway and its role in osseointegration.
- The effects of NSAIDs on osteoblasts attached to titanium are investigated (in vitro studies).

#### Data collection process

The full-text manuscripts of included studies were catalogued into in vitro, clinical, and in vivo studies. The data from the included studies were independently extracted by the primary (JDL) and the second reviewer (TJ) according to the “Data items” section as listed below. Disagreements or uncertainties were discussed with the third reviewer (MN) until an agreement was reached.

#### Data items

The data collected from the included studies were arranged in the following fields:

- Author (year)—reveals the author/s and year of publication
- Sample (size)—describes the sample and sample size used in the study
- Treatment group (size)—describes the treatments used in study
- Methodology—describes the method of drug delivery
- Parameter—describes the parameter/s that are measured
- Outcome—describes the outcome/s of the experiments

#### Quality and risk of bias in individual studies

The quality and risk of bias assessments were performed independently by two reviewers (JDL and TJ) during the data extraction process. Any disagreements or uncertainties were discussed with the third reviewer (MN) until an agreement was reached. The quality and bias assessment for all studies addressed various bias domains. A Modified CONSORT checklist of items for reporting in vitro studies of dental materials outlined by Faggion (Tables 1 and 2) was used to assess quality and risk of bias of included in vitro studies [16]. The Cochrane Collaboration’s Tool (Table 3) was used to assess quality and risk of bias of included human clinical studies [17]. A quality assessment of the methodology of the animal studies (Table 4) has been performed according to items (Table 5) of the ARRIVE guidelines [18].

**Table 1:** Quality assessment of in vitro studies according to the items of the Modified CONSORT checklist [16]

Study	1	2	3	4	5	6	7	8	9	10	11	12	13	14	Summary assessment
Arpornmaeklong et al. [19]	+	?	+	+	–	–	–	–	n/a	+	+	–	+	–	High
Boyan et al. [20]	+	+	+	+	–	–	–	–	n/a	+	+	–	+	–	High

Key: (+) = low risk of bias, (?) = unclear risk of bias, (−) = high risk of bias

**Table 2:** Modified CONSORT checklist of items for reporting in vitro studies of dental materials [16]

Item	Domain
1	Abstract: structured summary of trial design, methods, results, and conclusions
Introduction	
2	Scientific background and explanation of rationale with specific objectives and/or hypotheses
Methods	
3	Intervention: the intervention for each group, including how and when it was administered, with sufficient detail to enable replication
4	Outcomes: completely defined, pre-specified primary and secondary measures of outcome, including how and when they were assessed
5	Sample size: how sample size was determined
6	Randomisation: method used to generate the random allocation sequence
7	Allocation: mechanism used to implement the random allocation sequence, describing any steps taken to conceal the sequence until intervention was assigned
8	Implementation: who generated the random allocation sequence, who enrolled teeth, and who assigned teeth to intervention
9	Blinding: if done, who was blinded after assignment to intervention and how
10	Statistics: statistical methods used to compare groups for primary and secondary outcomes
Results	
11	For each primary and secondary outcome, results for each group, and the estimated size of the effect and its precision
Discussion	
12	Trial limitations, addressing sources of potential bias, imprecision, and, if relevant, multiplicity of analyses
Other information	
13	Sources of funding and other support role of funders
14	Where the full trial protocol can be accessed, if available

**Table 3:** Quality and bias assessment of human clinical studies using The Cochrane Collaboration’s Tool [17]

Study	Random sequence generation	Allocation concealment	Blinding of participants/personnel	Blinding of outcome assessment	Incomplete outcome data	Selective reporting	Summary assessment
Alissa et al. [21]	+	+	+	?	+	+	Unclear
Sakka et al. [24]	–	–	–	+	+	+	High
Winnett et al. [25]	n/a	–	–	–	?	+	High

Key: (+) = low risk of bias, (?) = unclear risk of bias, (−) = high risk of bias, *n/a= not available*

**Table 4:** Quality assessment of the methodology of the animal studies according to the items of the ARRIVE guidelines [18]

Study	5	6	7	8	9	10	11	12	13	Summary assessment
Cai et al. [22]	+	?	+	+	?	?	?	+	+	Unclear
Chikazu et al. [30]	+	–	+	+	–	–	?	+	+	High
Goodman et al. [29]	?	+	+	+	–	?	–	+	+	High
Goodman et al. [26]	?	+	+	+	–	?	–	+	+	High
Pablos et al. [31]	+	?	+	+	–	–	?	+	+	High
Ribeiro et al. [27]	+	?	+	+	–	–	?	+	+	High
Ribeiro et al. [28]	+	?	+	+	–	–	?	+	+	High
Salduz et al. [32]	–	+	+	+	?	–	+	+	+	High

Key: (+) = low risk of bias, (?) = unclear risk of bias, (−) = high risk of bias

**Table 5:** Items of the ARRIVE Guidelines [18]

Item	Domain
5	Ethical statement
6	Study design
7	Experimental procedures
8	Experimental animals
9	Housing and husbandry
10	Sample size
11	Allocating animals to experimental groups
12	Experimental outcomes
13	Statistical analysis

#### Synthesis of results

The relevant data collected for qualitative synthesis are summarised in three critical analysis tables: in vitro studies (Table 6), clinical studies (Table 7), and in vivo studies (Table 8).

**Table 6:** In vitro studies that investigated the effect of NSAIDs on osteoblasts attached to titanium surfaces

Study (year)	Sample	Treatment group	Methodology	Parameter	Outcome
Arpornmaeklong et al. (2009) [19]	Mouse calvaria cell line (MC3T3-E1)	Indomethacin 0.1 μM Celecoxib 1.5 μM Celecoxib 3.0 μM Celecoxib 9.0 μM Control	Incubation in treatment medium for 5 days. Investigations were performed in three experimental phases: static, log, and plateau	The following parameters were assessed at 1, 3, and 5 days: cell attachment, cell growth, cell differentiation, secretion of PGE2	Cells were able to grow and attach to titanium surface for all treatment groups. Indomethacin and celecoxib cell growth on days 3 and 5 in static phase and on day 3 in log phase. Indomethacin and celecoxib caused a significant decrease PGE2 concentration in static and plateau but not log phases.
Boyan et al. (2001) [20]	Human osteosarcoma cell line (MG63)	Indomethacin 0.1 μM Resveratrol 1 μM Resveratrol 10 μM NS-398 1 μM NS-398 10 μM	Incubation in treatment medium for 5 days. Cells were cultured on tissue culture plastic, smooth titanium, and two rough titanium surfaces: grit-blasted/acid-etched and titanium-plasma sprayed	The following parameters were assessed after 5 days: osteocalcin content, PGE2 content, and TGF-β1 content.	Indomethacin, resveratrol, and NS-398 had no effect on osteocalcin content. Indomethacin and resveratrol blocked PGE2 production. NS-398 had no effect on PGE2 production on smooth surfaces but caused a reduction on rough surfaces. Indomethacin blocked TGF-β1 production on rough surfaces. Resveratrol blocked TGF-β1 on TPS. NS-398 did not cause TGF-β1 inhibition.

**Table 7:** Clinical studies that investigated the effect of NSAIDs on osseointegration

Study (year)	Sample (size)	Treatment group (size)	Methodology	Parameter	Outcome
Alissa et al. (2009) [21]	Eligible human patients (*n* = 61). Implants inserted (*n* = 132).	Ibuprofen (*n* = 31), implants (*n* = 67). Placebo (*n* = 30), implants (*n* = 65).	Ibuprofen, 600 mg q.i.d. for 7 days orally	Post-operative radiographic marginal bone height at 3 and 6 months	No statistically significant differences in mean marginal bone level changes at 3 or 6 months.
Sakka et al. (2013) [24]	Eligible human patients (*n* = 28). Implants inserted (*n* = 57).	Ibuprofen (*n* = 14), implants (*n* = 31). Non-Ibuprofen (*n* = 14), implants (*n* = 26).	Ibuprofen, 600 mg q.i.d. for 7 days orally	Post-operative radiographic marginal bone height at 3 and 6 months	No statistically significant differences in mean marginal bone level changes at 3 or 6 months.
Winnett et al. (2014) [25]	Patients treated between 1979 and 2012 with failed and surgically removed dental implants	Cohort that used post-operative NSAIDs (*n* = 60, with 119 failed implants). Cohort that did not use post-operative NSAIDs (*n* = 44, with 78 failed implants).	Ibuprofen was the most commonly prescribed, 600 mg q.i.d. Other prescribed analgesics were ketorolac, vioxx, celebrex, diflunisal, meloxicam, paracetamol, and naproxen.	Radiographic bone loss. Vertical bone height of remaining implants.	NSAID cohort experienced more implant failures than the non-NSAID cohort. The NSAID cohort experienced more cases of radiographic bone loss greater than 30% of the vertical height of their remaining implants.

**Table 8:** In vivo studies using animal models that investigated the effect of NSAIDs on osseointegration

Study (year)	Sample (size)	Treatment group (size)	Methodology	Parameter	Outcome
Cai et al. (2015) [22]	New Zealand white rabbits (*n* = 18). Implant inserted into calvaria (*n* = 18).	Control (*n* = 6) Diclofenac (*n* = 6) Parecoxib (*n* = 6)	Treatments were administered for 7 days: Diclofenac, 2 mg/kg/day orally Parecoxib, 1.5 mg/kg/day subcutaneous injection	Parameters observed at week 4 and 12 after implantation: Micro-CT: bone volume ratio, mean trabecular thickness, and mean trabecular separation. Histomorphometric: bone-to-implant contact.	No statistically significant differences between the three separate groups, nor between the different time points.
Chikazu et al. (2007) [30]	9-week old male mice (*n* = 72). Implant inserted in femur (*n* = 72).	Mice with the original C57BL6/129S7 hybrid background were generated and maintained: Wild-type (*n* = 36) COX-2-knockout (*n* = 36)	No drug was administered	mRNA levels were observed at days 0, 1, 2, 4, 7, and 56 after implant insertion: *expression of COX-2 and osteocalcin mRNA*. Histomorphometric analysis at weeks 4 and 8: bone-to-implant contact.	Expression of COX-2 and osteocalcin mRNA was induced in bone surrounding implants in wild-type mice, but not in knockout mice. Bone-to-implant contact was minimal in knockout mice.
Goodman et al. (2002) [29]	New Zealand white rabbits (*n* = 8). Titanium bone harvest chamber inserted in tibia (*n* = 8).	Control Naproxen Rofecoxib	Treatments administered: Control: weeks 0–4 and 9–12. Naproxen, 110 mg/kg: weeks 5–8 orally. Rofecoxib, 12.5 mg/kg: week 13–16 orally.	Immunohistochemistry observed: total tissue area, total bone area, ratio of bone area, and total number of osteoblasts and osteoclast-like cells per section area	Naproxen and rofecoxib decreased bone ingrowth significantly. Rofecoxib decreased the area of osteoblasts per area compared with controls, and naproxen sodium did not reach statistical significance.
Goodman et al. (2005) [26]	New Zealand white rabbits (*n =* 8). Titanium bone harvest chamber implanted bilaterally in tibia (*n* = 16).	Control Rofecoxib	Treatments were administered for 6 weeks each: control-no drug; rofecoxib (12.5 mg/day) for the first 2 weeks of a 6-week trial, or the last 2 weeks or given continuously for all 6 weeks washout periods	Immunohistochemistry observed: total tissue area, total bone area, ratio of bone area, and the total number of osteoblasts and osteoclast-like cells per section area.	Rofecoxib given continuously for 6 weeks had less bone ingrowth, osteoclast-like cells and osteoblasts per area compared to the control treatment. Rofecoxib given for 2 of a 6-weeks cycle did not interfere with the parameters.
Pablos et al. (2008) [31]	Male Sprague-Dawley rats, 3-month-old, weighing 250-300 g (*n* = 30). Implant inserted in tibia (*n* = 30).	Control (*n* = 10) Diclofenac (*n* = 10) Meloxicam (*n* = 10)	Diclofenac, 1.07 mg/kg b.i.d. for 5 days. Meloxicam, 0.2 mg/kg daily for 5 days.	Histomorphometric analysis at 28 days after implant insertion: bone-to-implant contact, cortical bone area, and trabecular bone area within the implant threads	The bone-to-implant contact was lower in diclofenac compared with the meloxicam and control. The trabecular bone area was greater in diclofenac compared with meloxicam and control.
Ribeiro et al. (2006) [27]	Male Wistar rats, aged 10 weeks (*n* = 31). Implant inserted in tibia (*n =* 31).	Control (*n* = 14) Meloxicam (*n* = 17)	Daily subcutaneous injections for 60 days: control, 1 mL/kg of saline Meloxicam, 3 mg/kg	Histomorphometric analysis at 60 days after implant insertion: bone-to-implant contact, bone area, and bone density in the cortical and cancellous bone areas	Meloxicam reduced bone-to-implant contact, bone area, and bone density in both the cortical and cancellous bone areas.
Ribeiro et al. (2009) [28]	Male Wistar rats, aged 10 weeks (*n* = 30). Implant inserted in tibia (*n* = 30).	Control (*n* = 14) Meloxicam (*n* = 16)	Daily subcutaneous injections for 60 days: control, 1 mL/kg of saline Meloxicam, 3 mg/kg	Histomorphometric analysis at 60 days after implant insertion: bone-to-implant contact, bone area, and bone density in the cortical and cancellous bone areas	Blasting implant surface with aluminium oxide can increase bone-to-implant contact; however, it does not reverse the negative effects caused by a selective COX-2 inhibitor on bone healing around implants.
Salduz et al. (2017) [32]	New Zealand white rabbits, skeletally mature weighing 3.5–4 kg (*n* = 40). Titanium rods implanted bilaterally in femur (*n* = 80).	Control Diclofenac Celecoxib	Treatments were administered for 8 weeks: control, regular food Diclofenac, 5 mg/kg/day intramuscularly Celecoxib, 3 mg/kg/day orally	Biomechanical and histomorphometric analysis at 8 weeks after implant insertion: interface failure load, bone quality, bone implant interface, host reaction, total bone area, and bone-to-implant contact rate	No significant difference in the biomechanical and histological results between the groups.

#### Statistical analysis

No meta-analyses could be performed due to the heterogeneity between the studies—different samples, experimental groups (drugs and concentrations), study models, and outcome measures.

### Results

#### Study selection

**Fig. 1:**
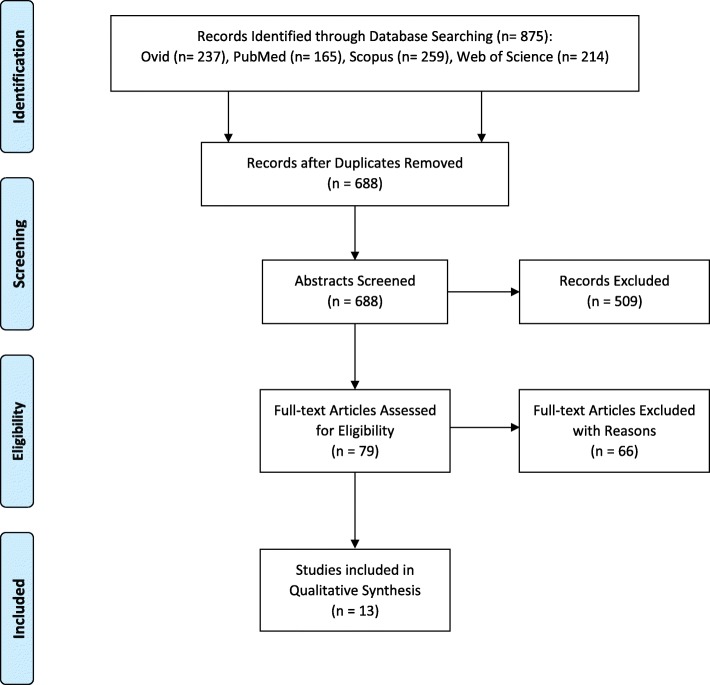
PRISMA flow diagram of literature search

The electronic search in the databases of Web of Science, PubMed, Ovid, and Scopus resulted in the identification of 875 potential titles and abstracts. After removal of the duplicates, independent screening of the abstracts resulted in the selection of 79 studies for assessment of eligibility. A total of 13 studies were eligible and are included in the systematic review (Fig. 1).

#### Exclusion of studies

The eligibility and study selection criteria as mentioned above were applied to the 79 full-text articles. A total of 66 studies were excluded after a full-text assessment for the following reasons:

- The study did not explore the role of COX pathway in osseointegration (*n* = 26).
- The effects of NSAIDs on osteoblasts were not investigated on titanium (*n* = 24).
- The study was a systematic review (*n* = 16).

#### Study characteristics

The included studies were catalogued into three groups characterised by the type of study: in vitro studies (Table 6), clinical studies (Table 7), and in vivo studies (Table 8). The cataloguing provided a clearer understanding of the effects of NSAIDs in osseointegration in various study models, ultimately contributing to the sensitivity of the systematic review.

#### Quality and risk of bias assessment

The quality and risk of bias assessments of included studies are summarised in Tables 1, 2, 3, 4, and 5. The quality assessment revealed a high risk of bias (for one or more domain) for most of the included studies. The included in vitro studies had high risk of bias according to the Modified CONSORT checklist [16, 19, 20]. One clinical study was classified as unclear risk of bias (for one or more domain) according to the Cochrane Collaboration’s Tool [17, 21]. An in vivo animal study had an unclear risk of bias according to the ARRIVE guidelines [18, 22].

### Discussion

Non-steroidal anti-inflammatory drugs are widely used in clinical dentistry to manage post-operative inflammation and pain. Two systematic reviews have been performed to review the literature concerning the possible influence of NSAIDs on the osseointegration of titanium implants: a review conducted by Gomes et al. concluded that osseointegration is impaired in the presence of conventional NSAIDs, whilst the review conducted by Kalyvas et al. concluded that short-term post-operative NSAIDs do not appear to negatively impact osseointegration [3, 4]. Despite these conflicting conclusions regarding post-operative use of NSAIDs, both Gomes et al. and Kalyvas et al. agreed that prolonged or long-term use of NSAIDs, particularly in patients with chronic diseases, impaired osseointegration and, therefore, reduced the success of implant surgery [3, 4]. The current review extends on these existing reviews by identifying if dosage, duration of administration, and selectivity of post-operative NSAIDs impair osseointegration.

#### In vitro studies

The effects of NSAIDs on the osteogenic activity of osteoblasts have been extensively studied at the molecular pharmacological level [23]. However, only two studies have been identified that investigated the effect of NSAIDs on osteoblasts attached to titanium surfaces (Table 6). In the study conducted by Boyan et al., their results demonstrated that a non-selective COX inhibitor (indomethacin, 0.1 μM), a selective COX-1 inhibitor (resveratrol, 1 and 10 μM), and a selective COX-2 inhibitor (NS-398, 1 and 10 μM) did not have a significant effect on the number of cells derived from human osteosarcomas [20]. Furthermore, Boyan et al. demonstrated that the NSAIDs reduced prostaglandin E_2_ (PGE2) production of cells attached to a rough titanium surface. Their results indicated that both COX-1 and COX-2 are involved in the production of osteocalcin, PGE2, and TGF-β1 by osteoblasts [20]. They also demonstrated that osteoblasts produced increased levels of PGE2 on rough titanium surfaces and that this was correlated with increased alkaline phosphatase activity and osteocalcin production [20]. This suggests that PGE2 may have a role in osteoblast proliferation and differentiation on titanium surfaces, and that this favourable effect of PGE2 was inhibited when a NSAID was present [20]. Arpommaeklong et al. found that a non-selective COXinhibitor (indomethacin, 0.1 μM) and a selective COX-2 inhibitor (celecoxib, 1.5, 3.0, and 9.0 μM) inhibited the growth of cell cultures derived from rat calvarias, where the effect was dose-dependent in the cultures treated with celecoxib [19]. Furthermore, Arpommaeklong et al. demonstrated that PGE2 levels were significantly lower in the groups that were treated with a NSAID, and have postulated that PGE2, consistent with Boyan et al., may have a role in osteoblast growth and differentiation [19, 20].

#### Clinical studies

The clinical evidence demonstrating the effects of NSAIDs on the osseointegration of titanium dental implants is limited with only two clinical trials and one retrospective study identified in the database searches (Table 7). In the clinical trial conducted by Alissa et al., the effect of a 7-day post-operative course of ibuprofen (600 mg, taken four times daily) on the marginal bone level around dental implants was investigated [21]. They found that there were no statistically significant differences between the treated and placebo groups in the mean marginal bone level around dental implants at 3 and 6 months after implant placement [21]. In a similar clinical trial conducted by Sakka et al., the effect of a 7-day course of ibuprofen (600 mg, taken four times daily) on the marginal bone level around dental implants was also investigated [24]. They found that there were no significant differences between the ibuprofen and non-ibuprofen groups, consistent with the findings of Alissa et al., when comparing the changes in marginal bone level around dental implants [24]. However, a retrospective study conducted by Winnett et al. postulated that the adverse biological events following dental implant placement were associated with perioperative use of NSAIDs [25]. Winnett et al. reported a total loss of 197 dental implants due to failed osseointegration from patients with failing implant/s (468 implants in 104 patients) treated in a postgraduate dental clinic (between 1979 and 2012). The patients (*n* = 60) that used NSAIDs peri-operatively experienced a total of 119 failed implants, whilst the non-NSAID cohort (*n* = 44) experienced a total of 78 failed implants. Winnett et al. identified that ibuprofen (600 mg, taken four times daily) was the most commonly prescribed; however, other prescribed NSAIDs included Ketorolac, Vioxx, Celebrex, Diflunisal, Meloxicam, and Naproxen [25]. Despite the clinical trials conducted by Alissa et al. and Sakka et al., both of whom have demonstrated that a 7-day post-operative course of ibuprofen (600 mg, taken four times daily) did not significantly affect bone levels around dental implants at 3 and 6 months after placement, the data gathered by Winnett et al. indicates that NSAIDs may have detrimental effect on osseointegration [25].

#### Animal studies

The influence of NSAIDS on bone healing in animal models has been shown to be related to the duration of treatment and drug selectivity [5]. A total of seven studies were identified that investigated the effect of NSAIDs on the osseointegration of titanium implants in animals: mice, rabbits, and rats (Table 8).

The duration of treatment is a factor to consider when using NSAIDs, and a study conducted by Goodman et al. investigated the effect of a selective COX-2 inhibitor (rofecoxib, 12.5 mg/kg/day) administered for 6 weeks on bone growth in a bone harvest chamber during three different time periods: initial 2 of the 6 weeks, final 2 of the 6 weeks, and continuously for 6 weeks [26]. The bone harvest chamber was a titanium device that was implanted into the tibia of rabbits and had an inner removable core with canals that allowed for bone ingrowth into the inner chamber. Their results revealed that rofecoxib given continuously for 6 weeks significantly reduced bone ingrowth and osteoblasts per area compared with the control (no treatment), whilst rofecoxib given for 2 weeks did not appear to interfere with bone ingrowth and number of osteoblasts [26]. Furthermore, the studies conducted by Ribeiro et al. investigated the effect of long-term administration (60 days) of a selective COX-2 inhibitor (meloxicam, 3 mg/kg/day) on the bone growth on a titanium implant [27, 28]. Their results also indicated that long-term use of a selective COX-2 NSAID significantly reduces bone-to-implant contact, bone area, and bone density, ultimately leading to failed osseointegration [27]. The data gathered in both studies suggest that duration of treatment is an important factor in the use of selective COX-2 NSAIDs, as short periods of rofecoxib and meloxicam did not adversely affect osseointegration [26, 27].

The COX selectivity of NSAIDs and their interference with prostaglandin synthesis have been shown to inhibit bone healing [23]. Goodman et al. performed a follow-up study where the titanium bone harvest chamber was again implanted into the tibia of rabbits and the rabbits were treated with water (control), a non-selective COX inhibitor (naproxen, 110 mg/kg/day), or a selective COX-2 inhibitor (rofecoxib, 12.5 mg/kg/day) for a 4-week period [29]. Their results again demonstrated that COX-2 inhibition significantly decreased bone ingrowth, where rofecoxib also decreased the number of osteoblasts per area [29]. Furthermore, the conclusions by Goodman et al. were supported by the study by Chikazu et al. where titanium implants were inserted in wild-type and COX-2-knockout mice [30]. Their results revealed that the expression of COX-2 was induced in bone surrounding the implants in wild-type mice, but not in COX-2-knockout mice and that the bone-to-implant contact was minimal in newly formed bone in COX-2-knockout mice [30]. The data collected by Goodman et al. and Chikazu et al. postulated that COX-2 may have an important role in osseointegration [26, 30]. However, a study conducted by Pablos et al., that investigated the effect of a non-selective COX inhibitor (diclofenac, 1.07 mg/kg/day) and a selective COX-2 inhibitor (meloxicam, 0.2 mg/kg/day) administered for 5 days on peri-implant healing in rats, revealed that diclofenac delayed peri-implant bone healing and negatively affected the bone-to-implant contact, whereas meloxicam had no negative effect on peri-implant healing [31]. The results of Pablos et al. were inconsistent with the results of the study conducted by Cai et al. that also investigated the effect of diclofenac (2 mg/kg/day) and a selective COX-2 inhibitor (parecoxib, 1.5 mg/kg/day) administered for 7 days on the osseointegration of titanium implants in rabbit calvarias. Their results revealed no statistically significant differences between the experimental groups and the control [22]. Furthermore, a recent study conducted by Salduz et al. that investigated the effect of a non-selective COX inhibitor (diclofenac, 5 mg/kg/day) and a selective COX-2 inhibitor (celecoxib, 3 mg/kg/day) administered for 8 weeks on bone growth and osseointegration on two different alternative titanium surfaces revealed no statistically significant differences in the biomechanical and histological results between the experimental groups and the control, suggesting that long-term use of NSAIDs does not affect osseointegration [32]. The data collected in animal studies regarding duration of treatment and drug selectivity is inconsistent, and there is a lack of consensus on the influence of NSAIDs on osseointegration in animal models.

#### Limitations

The majority of the included studies revealed a high risk of bias, and conclusions from studies that have a high risk of bias are sufficient to affect interpretation of data [16–18]. Publication and selection bias is apparent in several included studies, as the negative effects of NSAIDs on osseointegration can be expected in the studies that administered NSAID at a high concentration and/or for a prolonged period of time. The conclusions of this systematic review were largely based on animal studies, as there are very few published in vitro and clinical studies relating to the effect of NSAIDs on osseointegration. The effects of NSAIDs on osseointegration in animals cannot be translated to humans due to the vastly different pharmacokinetics.

## Conclusions

The analgesic and therapeutic effects of NSAIDs are achieved by COX-2 inhibition [4]. It is likely that COX inhibition by NSAIDs is detrimental to the bone healing process, given the favourable actions of PG on this process [4]. Osteoblasts have the capacity to produce PGs, where PGE2 is most abundant, through the COX pathway though the evidence asserting that PGs have a direct role in bone healing is inconclusive [1, 23]. Furthermore, there is insufficient evidence in the current literature to explicitly conclude that there is a relationship between the use of NSAIDs and early implant failure. However, osseointegration does not appear to be negatively affected by NSAIDs in the human clinical studies, which contrasts with the experimental in vitro and in vivo animal studies. Furthermore, there are no human clinical studies that have investigated the effect of a selective COX-2 NSAID on osseointegration. Therefore, further research with an emphasis in human clinical studies comparing the effect of the COX selectivity of NSAIDs on osseointegration is required.

## Abbreviations

COX: Cyclooxygenase
NSAID/s: Non-steroidal anti-inflammatory drug/s
PG: Prostaglandin
PGE2: Prostaglandin E2
